# 3D-Printing of zirconia dental prostheses

**DOI:** 10.1080/07853890.2021.1897420

**Published:** 2021-09-28

**Authors:** I. Rodrigues, S. Olhero, M. Guedes, A. P. Serro, C. G. Figueiredo-Pina

**Affiliations:** a CDP2T and Department of Mechanical Engineering, School of Technology of Setúbal, Instituto Politécnico de Setúbal, Portugal; b CeFEMA, Center of Physics and Engineering of Advanced Materials, Instituto Superior Tecnico, Universidade de Lisboa, Lisboa, Portugal; c Department of Materials and Ceramics Engineering, CICECO, University of Aveiro, Aveiro, Portugal; d CQE, Instituto Superior Tecnico, Universidade de Lisboa, Lisboa, Portugal; e Centro de Investigação Interdisciplinar Egas Moniz (CiiEM), Egas Moniz Cooperativa de Ensino Superior, Caparica, Portugal

## Abstract

**Introduction:**

Yittria-stabilised-zirconia is a biomaterial widely employed in the production of dental prostheses through several traditional manufacturing and shaping processes (such as machining and surface grinding of a green or sintered block, gel-casting and injection moulding) which carry relatively high labour, tooling, moulding and waste material costs. Additive manufacturing 3D-printing processes, such as robocasting, enable the quick production of customised and intricate parts without the need for costly milling tools and moulds [1,2]. In this work, the robocasting process was employed in the production of zirconia parts using a layer-by-layer deposition strategy of a dispersed ceramic powder slurry through a numerically controlled extruder and nozzle. The highest possible solid content allowing for proper extrusion through a fine nozzle was determined for a plain, inexpensive water-based slurry of zirconia powders and dispersing agent.

**Materials and methods:**

A commercial open-source 3D printer was modified to use a standard micrometric syringe and needle as an extruder Figure 1. An initial batch of 80 wt.% solid zirconia powder slurry was produced using a predetermined optimal CE64 dispersant ratio and equal portions were subsequently condensed through air-drying at 50 °C until an 82, 84, 86, 88 or 90 wt.% solid content ratio was reached. Each slurry was then used for the robocasting of zirconia cuboid samples, which were air-dried for 48 h at 50 °C and sintered at 1500 °C for 2 h. Sintered sample densities were measured using the Archimedes method and the polished base sample surfaces were characterised through microscopy analysis, hardness and toughness tests. The slurry yielding the best results was, thus selected for the production of a sintered tooth prototype using the same sample extrusion speeds, nozzle travel speeds and diameter (0.45 mm).

**Results:**

The best sample print quality was attained using 88 wt.% solid content slurries, which resulted in minimal microscopic sintering defects and no extrusion faults due to transient clogging of the extrusion nozzle, thus reaching a sintered density 97% that of theoretical. A full-size tooth prototype was successfully fabricated using the 88 wt.% solid content slurries Figure 2. **Discussion and conclusions:** The 88 wt.% solid content slurry showed increased viscosity, consistently raising extrusion pressure and shear stresses which likely broke-up any soft agglomerates, thus avoiding temporary nozzle clogging and the formation of agglomerate-related sintering defects. The 90 wt.% slurry showed near-dilatant behaviour, possibly possessing harder agglomerates. Thus, sintered sample parts with 97% theoretical density were realised from an 88 wt.% plain water-based slurry with no binders or plasticisers that would otherwise require an additional pre-sintering burnout stage and likely generate sintering defects.

**Figure 1.**
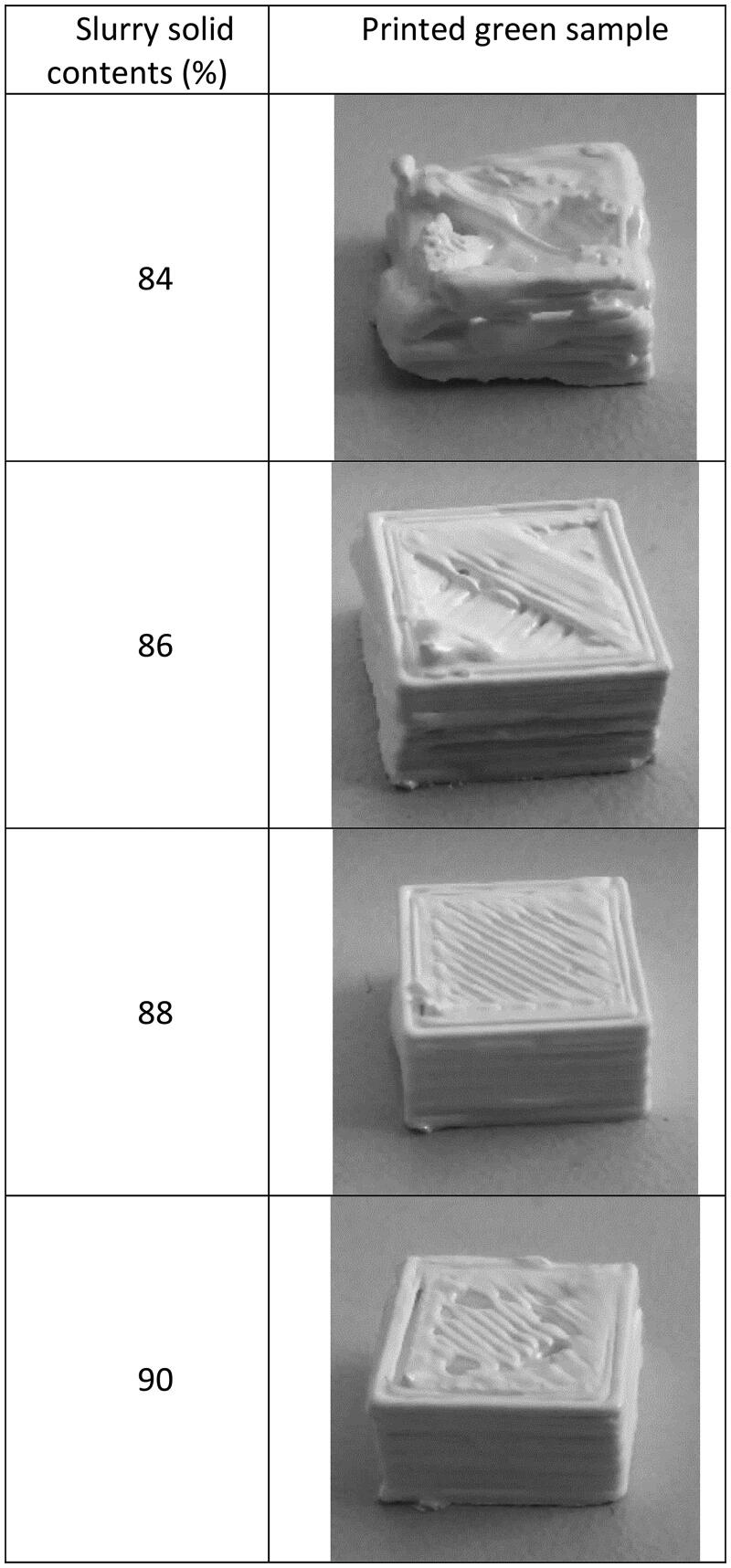
Visual comparison of green samples robocasted from printable pastes.

**Figure 2.**
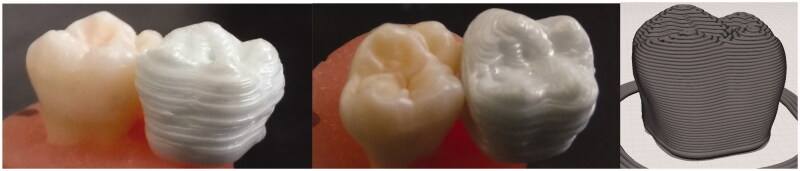
Post-sintering public domain molar tooth prototype printed using the using the 88 wt.% solid slurry next to an unrelated extracted tooth for comparison. The sliced 3D prototype tooth model used is shown in the rightmost picture.
